# Natural Killer T cells – balancing the regulation of tumor immunity

**DOI:** 10.1038/bjc.2012.453

**Published:** 2012-11-06

**Authors:** M W Lowdell

**Affiliations:** 1 Department of Haematology, Royal Free Hospital, UCL, London, UK

Natural killer T (NKT) cells are one of the minor lymphoid subsets that, similar to *γ*/*δ* T cells and NK cells, are emerging as essential factors in the linkage of innate and acquired immunity. This book provides a detailed and comprehensive review of the considerable basic science behind our understanding of the biology of these cells and their role in tumour immunology. The final chapters cover the clinical trials of NKT-activating agents and of adoptive anti-tumour immunotherapy with NKT.

The editors have put together a first-class team of experts to write each chapter, and the arrangement of their submissions provides a logical progression so that the book is actually greater than the sum of its parts. A persistent criticism is that the editors have, at times, failed to be sufficiently aggressive and allowed the introduction to basic NKT biology to be repeated by several contributors.

Readers who are new to the NKT field should read the Preface as it provides the only early explanation: the NKT cells are divided into type I and type II subsets. Most NKT research has addressed the type I subset, those which all express the Vα14 Jα18 TCR and recognise a very restricted variety of antigens presented by CD1d; the so-called invariant NKT cells (iNKT). The rest of the first half of the book focuses almost exclusively on iNKT cells.

The opening chapter defines the iNKT cell and reviews the history of their discovery. It goes on to provide a detailed explanation of the ligands involved in iNKT activation and their role in anti-tumour immunity. The chapter ends with an introduction to iNKT priming for therapeutic use and the induction of iNKT from induced pluripotent stem cells (iPS), which is a specific interest of the chapter authors. This is the only part of the book that feels misplaced, being too esoteric for a book chapter. The clinical use of iNKT priming is better covered in the penultimate chapter and the review of iPS-derived iNKT would have been better placed within the final chapter on the clinical use of iNKT.

Antigen recognition by iNKT cells is the subject of Chapter 2. This is a tour-de-force of biochemistry, which certainly overwhelmed this reviewer. The introduction includes significant repetition of information in Chapter 1, which could have been edited and the style is more like a PhD thesis than a book chapter in some places with clumsy linkage of information forming non-sequiturs. The biochemical detail of the antigens is possibly too much for a text book. It is particularly surprising that, in a book about tumour immunity, there is no discussion about how the microbial and self-antigens recognised by iNKT might be physiologically involved in tumour immunity or, indeed the search for tumour-derived, CD1d-restricted antigens.

The remaining chapters firmly establish this as a text book on the potential clinical use of iNKT in tumour immunotherapy starting with a very readable chapter on iNKT vaccine strategy, which leads very smoothly into a review of NKT cell regulation of innate and acquired tumour immunity. This is the first chapter to cover type II NKT and explain their interaction with type I NKT and other immune cells and is an excellent review. It is followed by a more detailed explanation of tumour recognition by type I and type II NKT and includes a section on type II NKT recognition of CD1d-negative tumours. This chapter closes with a review of our knowledge of NKT-evasion strategies by tumours, which is the best evidence of the importance of these cells in natural tumour surveillance.

It is apparent by this stage of the book that iNKT and NKT are, together with NK and *γ*/*δ* T cells, central to the innate=acquired immunity axis and that dendritic cells are the ‘roundabout’ at which these two roads meet and interact. Chapter 6 covers the clinical use of iNKT and iNKT-Ags to induce dendritic cell maturation, interact with NK cells and induce CD4 and CD8 T-cell responses to tumour antigens. It is followed by a review of potential anti-tumour therapies based on type I and type II NK cells, which sadly repeats much of the information in Chapter 5 and would have benefited from more editing.

A theme introduced in Chapter 1 was the relative difficulty of isolating iNKT from cancer patients for therapeutic use and the potential solution of deriving the cells from iPS. The quantitative and qualitative deficiencies of iNKT from cancer patients is covered in Chapter 8, which once again opens with another introduction to type I and type II NKT and their role in initiation of conventional T-cell responses, which is highly repetitive. The chapter provides the first review of early clinical trials in solid tumours with iNKT and iNKT-activating antigens and leads into the following chapter focussing on NKT cell responses to haematological malignancies. The book concludes with two chapters presenting results from and summarising clinical trials including those involving adoptive autologous immunotherapy with *ex vivo* expanded iNKT.

This is a substantial review of current knowledge and understanding of the biology and function of type I and type II NKT cells and their potential clinical application in cancer therapy. It would benefit from removal of repetitive introductions. The references are extremely comprehensive throughout but are presented as first author plus publication year in parentheses throughout the text, which disrupts the flow and would be improved by using a less intrusive numbered reference system. Nonetheless, this is a comprehensive, readable and remarkably up to date text book that reflects the dynamism of the field and forms a useful reference work.

